# Supplementary material for: Understanding the contribution of public- and restricted-access places to overall and domain-specific physical activity among Mexican adults: A cross-sectional study
Source: PLoS One. 2020 Feb 7;15(2):e0228491. doi: 10.1371/journal.pone.0228491 (PMC7006922; doi:10.1371/journal.pone.0228491)
Supplement: S1 Table — (DOCX) [file pone.0228491.s001.docx]

**S1 Table. Most frequently reported places for physical activity (PA) among males and females from Mexico (n=3,686).**

|  | **Male** | | **Female** | |
| --- | --- | --- | --- | --- |
| **Places for PA** | **Ranking** | **% (95% CI)^c^** | **Ranking** | **% (95% CI)^c^** |
| **Public or private** |  |  |  |  |
| Any (≥1) public-  access place |  | 83.6 (81.7, 85.4) |  | **73.8 (71.3, 76.2)** |
| Any (≥1) restricted-  access place |  | 39.2 (36.8, 41.2) |  | **31.9 (29.4, 34.5)** |
| Any (≥1) place |  | 94.2 (92.9, 95.3) |  | **87.0 (84.9, 88.7)** |
| **Specific places** |  |  |  |  |
| Home | 3 | 35.6 (33.2, 38.1) | 1 | **50.5 (47.8, 53.3)** |
| Parks ^a^ | 1 | 45.6 (43.0, 48.1) | 3 | **36.3 (33.7, 38.9)** |
| Streets ^a^ | 2 | 41.1 (38.6, 43.7) | 2 | 37.9 (35.2, 40.6) |
| Open green spaces ^a^ | 4 | 26.8 (24.6, 29.1) | 4 | 23.3 (21.0, 25.7) |
| Shopping malls ^a^ | 7 | 20.2 (18.2, 22.4) | 5 | 22.5 (20.1, 25.0) |
| Private gyms ^b^ | 6 | 22.0 (20.1, 24.1) | 6 | 19.9 (17.8, 22.1) |
| Work | 8 | 17.3 (15.5, 19.3) | 7 | **14.2 (12.3, 16.3)** |
| Outdoor court ^a^ | 5 | 22.8 (20.8, 25.0) | 9 | **8.1 (6.8, 9.6)** |
| Plazas ^a^ | 9 | 14.3 (12.3, 16.1) | 8 | **10.0 (8.5, 11.8)** |
| Private sports facilities ^b^ | 10 | **9.8 (8.4, 11.4)** | **11** | **5.7 (4.5, 7.1)** |
| School/University ^b^ | 12 | 8.5 (7.3, 9.9) | 10 | 6.7 (5.5, 8.1) |
| Cycling path ^a^ | 11 | 8.7 (7.4, 10.2) | 12 | **4.6 (3.6, 5.8)** |
| Indoor courts ^a^ | 13 | 5.9 (4.9, 7.1) | 13 | **3.1 (2.3, 4.1)** |
| Bars & night clubs ^b^ | 14 | 3.6 (2.8, 4.6) | 14 | 2.4 (1.8, 3.2) |
| Other | 16 | 1.6 (1.1, 2,5) | 15 | 2.7 (1.8, 4.0) |
| Museums ^b^ | 15 | 2.0 (1.4, 2.7) | 16 | 1.8 (1.1, 2.8) |

Ranking. 1=most prevalent

a. Open-access place: no cost, membership or affiliation may be required for access and use.

b. Restricted-access place: cost, membership or affiliation required for access and use. Excludes home and work.

c. Estimations (% and 95% CI) are weighted using post-stratification survey weights

**Bolds** indicate significantly different (p<0.01) from males
