# Supplementary material for: Understanding the contribution of public- and restricted-access places to overall and domain-specific physical activity among Mexican adults: A cross-sectional study
Source: PLoS One. 2020 Feb 7;15(2):e0228491. doi: 10.1371/journal.pone.0228491 (PMC7006922; doi:10.1371/journal.pone.0228491)
Supplement: S2 Table — (DOCX) [file pone.0228491.s002.docx]

**S2 Table. Most frequently reported places for physical activity (PA) across education levels among adults from Mexico (n=3,686).**

|  | **Less than high school** | | **Undergraduate** | | **Graduate** | |
| --- | --- | --- | --- | --- | --- | --- |
| **Places for PA** | **Ranking** | **% (95% CI)^c^** | **Ranking** | **% (95% CI)^c^** | **Ranking** | **% (95% CI)^c^** |
| **Public or private** |  |  |  |  |  |  |
| Any (≥1) public-  access place |  | 76.3 (72.3, 79.9) |  | 79.4 (77.5, 81.2) |  | 77.1 (72.5, 81.1) |
| Any (≥1) restricted-  access place |  | 33.2 (29.3, 37.3) |  | 34.6 (32.6, 36.8) |  | 41.5 (36.5, 46.6)^d,e^ |
| Any (≥1) place |  | 87.5 (84.2, 90.2) |  | 91.0 (89.6, 92.3) |  | 91.0 (87.3, 93.7) |
| **Specific places** |  |  |  |  |  |  |
| Home | 1 | 49.0 (44.6, 53.4) | 1 | 43.9 (41.7, 46.2) | 3 | 34.2 (29.4, 39.4) ^d,e^ |
| Parks ^a^ | 2 | 41.5 (37.3, 45.8) | 2 | 40.1 (37.9, 42.3) | 1 | 43.0 (38.0, 45.8) |
| Streets ^a^ | 3 | 40.1 (35.9, 44.5) | 3 | 39.8 (37.6, 42.1) | 2 | 36.8 (32.0, 41.9) |
| Open green spaces ^a^ | 4 | 23.8 (20.3, 27.7) | 4 | 25.2 (23.3, 27.2) | 5 | 25.2 (21.0, 30.0) |
| Shopping malls ^a^ | 5 | 20.9 (17.4, 24.9) | 5 | 21.6 (19.7, 23.6) | 6 | 21.1 (17.0, 25.8) |
| Private gyms ^b^ | 7 | 16.4 (13.5, 19.8) | 6 | 20.8 (19.1, 22.6)^d^ | 4 | 26.7(22.5, 31.3)^d,e^ |
| Work | 8 | 15.2 (12.4, 18.5) | 7 | 16.2 (14.6, 18.0) | 7 | 13.7 (10.5, 17.7) |
| Outdoor court ^a^ | 6 | 17.9 (14.9, 21.3) | 8 | 15.3 (13.9, 16.9) | 9 | 11.5 (8.6, 15.2 )^d,e^ |
| Plazas ^a^ | 9 | 12.0 (9.4, 15.1) | 9 | 11.9 (10.5, 13.4) | 8 | 13.2 (10.1, 17.1) |
| Private sports facilities ^b^ | 11 | 7.8 (5.7, 10.5) | 10 | 7.7 (6.5, 9.0) | 11 | 7.6 (5.4, 10.7) |
| School/University ^b^ | 10 | 10.5 (8.3, 13.1) | 11 | 6.7 (5.7, 7.8) ^d^ | 10 | 8.5 (5.8, 12.2)^d^ |
| Cycling path ^a^ | 12 | 5.8 (4.1, 8.0) | 12 | 6.6 (5.7, 7.8) | 12 | 7.2 (5.0, 10.2) |
| Indoor courts ^a^ | 13 | 5.2 (3.8, 7.2) | 13 | 4.5 (3.8, 5.5) | 14 | 3.0 (1.7, 5.1) |
| Bars & night clubs ^b^ | 14 | 3.2 (2.2, 4.8) | 14 | 3.0 (2.4, 3.7) | 15 | 2.6 (1.5, 4.4) |
| Other | 15 | 1.4 (0.7, 3.1) | 15 | 2.4 (1.6, 3.3) | 16 | 2.3 (1.2, 4.4) |
| Museums ^b^ | 16 | 0.03 (0.01,0.1) | 16 | 1.9 (1.3, 2.6) | 13 | 3.4 (1.9, 6.0)^d^ |

Ranking. 1=most prevalent

a. Open-access place: no cost, membership or affiliation may be required for access and use.

b. Restricted-access place: cost, membership or affiliation required for access and use. Excludes home and work.

c. Estimations (% and 95% CI) are weighted using post-stratification survey weights

d. Significantly different (p<0.01) from less than high school

e. Significantly different (p<0.01) from undergraduate
