# Supplementary material for: Understanding the contribution of public- and restricted-access places to overall and domain-specific physical activity among Mexican adults: A cross-sectional study
Source: PLoS One. 2020 Feb 7;15(2):e0228491. doi: 10.1371/journal.pone.0228491 (PMC7006922; doi:10.1371/journal.pone.0228491)
Supplement: S3 Table — (DOCX) [file pone.0228491.s003.docx]

**S3 Table. Most frequent places for physical activity across categories of income adequacy* among Mexican adults (n=3,686).**

|  | **Difficult** | | **Neither easy nor difficult** | | **Easy** | |
| --- | --- | --- | --- | --- | --- | --- |
| **Places for PA** | **Ranking** | **% (95% CI)^c^** | **Ranking** | **% (95% CI)^c^** | **Ranking** | **% (95% CI)^c^** |
| **Private or public** |  |  |  |  |  |  |
| Any (≥1) public-  access place |  | 79.3 (76.8, 81.6) |  | 78.8 (76.1, 81.2) |  | 76.4 (72.7, 79.8) |
| Any (≥1) restricted-  access place |  | 27.8 (25.3, 30.5) |  | 38.6 (35.8, 41.5) ^d^ |  | 45.0 (41.0, 49.1)^d^ |
| Any (≥1) place |  | 89.1 (87.1, 90.8) |  | 91.2 (89.1, 92.9) |  | 91.9 (89.1, 93.4) |
| **Specific places** |  |  |  |  |  |  |
| Home | 1 | 44.3 (41.4, 47.3) | 1 | 44.6 (41.6, 47.6) | 2 | 39.0 (35.0, 43.0) |
| Parks ^a^ | 3 | 39.4 (36.6, 42.4) | 2 | 42.0 (39.1, 45.0) | 1 | 40.9 (37.0, 45.0) |
| Streets ^a^ | 2 | 42.9 (39.9, 45.9) | 3 | 38.3 (35.4, 41.3) | 3 | 34.4 (30.7, 38.5)^d^ |
| Open green spaces ^a^ | 4 | 22.6 (20.2, 25.1) | 4 | 27.9 (25.2, 30.7)^d^ | 5 | 24.4 (21.1, 28.1) |
| Shopping malls ^a^ | 5 | 21.6 (19.2, 24.2) | 5 | 22.6 (20.1, 25.5) | 6 | 18.5 (15.4, 21.9) ^d^ |
| Private gyms ^b^ | 7 | 15.0 (13.1, 17.2) | 6 | 22.3 (20.0, 24.8)^d^ | 4 | 30.5 (27.0, 34.3) ^d,e^ |
| Work | 6 | 16.3 (14.2, 18.6) | 8 | 15.5 (13.4, 17.9) | 7 | 14.8 (12.0, 18.0) |
| Outdoor court ^a^ | 8 | 14.7 (12.8, 16.8) | 7 | 16.0 (14.0, 18.2) | 8 | 14.6 (12.1, 17.5) |
| Plazas ^a^ | 9 | 11.0 (9.3, 13.0) | 9 | 12.6 (10.7, 14.8) | 9 | 13.3 (10.8, 16.2) |
| Private sports facilities ^b^ | 11 | 5.6 (4.4, 7.1) | 10 | 9.0 (7.4, 10.8) ^d^ | 10 | 9.5 (7.2, 12.4) ^d^ |
| School/University ^b^ | 10 | 6.4 (5.2, 7.7) | 11 | 8.7 (7.2, 10.5) | 12 | 8.0 (6.0, 10.5) |
| Cycling path ^a^ | 12 | 4.8 (3.7, 6.2) | 12 | 7.4 (6.1, 9.0) ^d^ | 11 | 8.7 (6.8, 11.1) ^d^ |
| Indoor courts ^a^ | 13 | 3.6 (2.7, 4.7) | 13 | 5.4 (4.3, 6.7) | 13 | 4.4 (3.1, 6.1) |
| Bars & night clubs ^b^ | 15 | 2.5 (1.8, 3.49 | 14 | 3.4 (2.5, 4.4) | 14 | 3.2 (2.1, 4.8) |
| Other | 14 | 3.1 (2.1, 4.5) | 15 | 1.8 (1.1, 3.1) | 16 | 0.9 (0.4, 2.3) |
| Museums ^b^ | 16 | 1.4 (0.9, 2.1) | 16 | 1.8 (1.1, 2.8) | 15 | 3.2 (1.9, 5.3) |

Ranking. 1=most prevalent

a. Open-access place: no cost, membership or affiliation may be required for access and use.

b. Restricted-access place: cost, membership or affiliation required for access and use. Excludes home and work.

c. Estimations (% and 95% CI) are weighted using post-stratification survey weights

d. Significantly different (p<0.01) from difficult

e. Significantly different (p<0.01) from neither easy nor difficult
