# Supplementary material for: Understanding the contribution of public- and restricted-access places to overall and domain-specific physical activity among Mexican adults: A cross-sectional study
Source: PLoS One. 2020 Feb 7;15(2):e0228491. doi: 10.1371/journal.pone.0228491 (PMC7006922; doi:10.1371/journal.pone.0228491)
Supplement: Attachment — Submitted filename: Reviewer comments 1 PONE.docx [file pone.0228491.s004.docx]

PONE-D-19-30067
Understanding the contribution of public- and restricted-access places to overall and domain-specific physical activity among Mexican adults: A cross-sectional study.

Reviewer #1: The manuscript is well-written, very concise and informative for providing evidence to inform the design of public strategies. There are some minor comments and observations for the paper. The discussion is very well-developed and it is easy to follow. This section adds relevant and contextual information about the implications of these findings.

Introduction.
L77. In the paper is mentioned that a limitation of current studies is that they only included one city from each studied country… could you provide in methods (maybe in the discussion too) briefly more details about the included cities in the study. It could be relevant for international readers.

Response: Thank you for your suggestion. We have now detailed the cities included in each of the three countries in the introduction and in the discussion sections (L77-78 and L272-274).

Methods.
L110. Please check the reference, as the criterion validity was not tested in Brazil. Also, in general, the paper describes that reliability was moderate and validity poor to fair in other countries.

Response: We thank the reviewer for pointing out this mistake. We have now included addittional references reporting on the criterion validity of GPAQ among Latinamerican populations and corrected the sentence reporting the validity of the GPAQ to reflect that this instrument has poor to fair validity (L112-113) .

L114. Add at the end of the sentence in the parenthesis… “per week of MVPA).”

Response: Done

Statistical analysis. In the abstract, you mentioned the analytical method used in this paper, but it was not mentioned in this section.

Response: We thank the reviewer for identifying this omission. We have now included in this section the type of models used (i.e. multivariate logistic regression models) (L163).

Results
How did you evaluate implausible or inconsistent results? (you may add details in methods).

Response: Implausible and inconsistent PA data was evaluated according to the GPAQ protocol. We have now included additional information and examples of how this was achieved in the “Physical activity measures” section (L114-116).

L181. For international comparability, you may use the term university instead of college as in some countries that refer to high school.

Response: Done

L213. minor detail. use PA instead of physical activity.

Response: Corrected

L225. minor detail. add % after 29 to be consistent.

Response: Corrected

Discussion
In the discussion, please describe the implications of having a more educated sample in this study compared with the Mexican population. You may add some examples or hypothesise something. I know you highlight this in the limitations (L347).

Response: Wee thank the reviewer for the suggestion. We have now included additional information relating the implications of having a more educated sample in the study compared with the Mexican popualtion (L287-290).

Tables and figures
I may include the supplementary tables in the paper as you described them in the results. It would be very useful to see the sex differences in the full version of the manuscript, for example.

Response: We thank the reviewer for the suggestion. We have now included an additional figure showing differences by demographic characteristics (i.e. sex, education and income adequacy) (see Figure 2, L218-220). We decided to keep supplementary tables (i.e. S1, S2 and S3 Table) because they provide additional information which may be useful for readers (L210-211).
